# Trans-Endometrial Cesarean Myomectomy for a Large Anterior Lower-Segment Intramural Fibroid Preventing Hysterotomy Closure

**DOI:** 10.14740/jmc5308

**Published:** 2026-04-29

**Authors:** Monchai Suntipap, Potsanop Kassayanan, Kasidis Nontaprom

**Affiliations:** a Department of Obstetrics and Gynecology, Faculty of Medicine, Srinakharinwirot University, Ongkharak, Nakhon Nayok 26120, Thailand; b Faculty of Medicine, Srinakharinwirot University, Ongkharak, Nakhon Nayok 26120, Thailand

**Keywords:** Cesarean delivery, Cesarean myomectomy, Hysterotomy closure, Intramural fibroid, Lower uterine segment, Trans-endometrial myomectomy, Uterine fibroid

## Abstract

Cesarean myomectomy has traditionally been avoided because of hemorrhage risk, but removal at cesarean delivery (CD) may be required when a fibroid distorts the lower uterine segment or prevents secure hysterotomy closure. A 35-year-old primigravida with fetal growth restriction at 38 + 2 weeks’ gestation had a large anterior intramural fibroid in the lower uterine segment. Intraoperatively, the fibroid caused distortion and traction along the hysterotomy line, and prevented approximation of the myometrial edges, precluding secure closure without excision. A trans-endometrial myomectomy was performed. The lesion was enucleated using combined blunt and sharp dissection, and the myoma bed was repaired with targeted hemostatic suturing. Hemostasis was supported with continuous intravenous oxytocin infusion, followed by multilayer uterine closure. Estimated blood loss was 1,300 mL and one unit of packed red blood cells was transfused. Histopathology confirmed leiomyoma. At 6 weeks postpartum, the patient reported no secondary postpartum hemorrhage, fever, or pelvic pain, and transvaginal ultrasonography was normal. This case highlights that a trans-endometrial approach can be considered in selected situations, particularly when a large lower-segment intramural fibroid prevents secure uterine closure during CD.

## Introduction

Uterine fibroids are common benign smooth muscle tumors. Although most patients are asymptomatic, fibroids can cause pelvic pain, abnormal uterine bleeding, and infertility [1]. Fibroids in pregnancy have been associated with several adverse obstetric outcomes, including preterm birth, malpresentation, second trimester pregnancy loss, and labor obstruction [2]. In pregnancy, the prevalence of uterine fibroids estimated at approximately 9.6% [3]. Although the reported estimate was low, the actual burden of uterine fibroids in pregnancy may be higher in current practice because pregnancies increasingly occur at advanced maternal age and assisted reproductive technologies may allow more women with pre-existing fibroids to conceive. However, the burden may still be underestimated due to limited ultrasonographic detectability during gestation, regression of lesions, or incidental identification only during cesarean delivery (CD) [3, 4].

Cesarean myomectomy (CM) remains controversial. Traditional teaching discourages myomectomy at the time of CD except in selected circumstances, such as pedunculated fibroids, largely because of concerns about intraoperative hemorrhage [5]. However, although there is no definite indication for CM, recent evidence suggests that CM can be performed in carefully selected patients by experienced surgeons, although it may be associated with prolonged operative time and increased intraoperative blood loss compared with CD alone [6–8]. CM is generally avoided in pregnant women with a high risk of bleeding or when the fibroid is located close to major pelvic vessels [9]. Two main surgical approaches have been described: the traditional trans-serosal technique and the newer trans-endometrial approach, which is associated with reduced bleeding and fewer adhesions than the traditional technique [10–12]. Lower-segment fibroids present particular technical challenges at CD, including an increased risk of inadvertent bladder injury and lateral extension of the hysterotomy into the uterine vessels, which must be considered when planning the operative approach.

This case report describes a lower-segment anterior intramural fibroid located along the hysterotomy line that prevented secure uterine closure at CD, and required a trans-endometrial CM. The postoperative course was uncomplicated, and 6-week postpartum transvaginal ultrasonography was normal.

## Case Report

**Figure 1:**
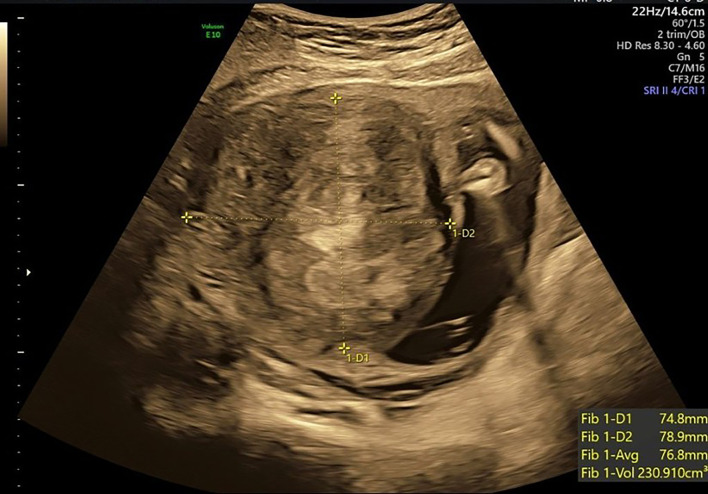
Prenatal ultrasound at 20 weeks’ gestation showing an anterior-wall intramural fibroid (yellow cross-marks) measuring approximately 7.5 × 7.9 cm. The lesion was in the lower anterior uterine segment.

A 35-year-old primigravida with gestational diabetes mellitus, managed with dietary modification and good glycemic control, presented at 38 + 2 weeks’ gestation with fetal growth restriction (FGR). At 20 weeks’ gestation, ultrasound examination revealed a large anterior intramural fibroid in the lower segment, measuring 7.5 × 7.9 cm (Fig. 1). At 38 weeks’ gestation, ultrasound assessment demonstrated an estimated fetal weight of 2,374 g (below the 10th percentile) with normal umbilical artery findings. The fibroid measured 5.9 × 7.6 cm in the right lower uterine segment; the apparent reduction in dimensions likely reflected measurement artifact attributable to restricted acoustic windows at late gestation, rather than true fibroid regression.

Following multidisciplinary discussion that considered FGR, term gestation, and the lower-segment fibroid, labor induction was planned. The fibroid was located eccentrically in the right lower uterine segment and, given the relatively small estimated fetal weight, was not anticipated to mechanically obstruct labor or vaginal delivery. Although the fibroid’s proximity to the lower uterine segment was recognized as a potential source of hemorrhage at CD, preoperative assessment suggested that fetal delivery could be achieved by maneuvering around the lesion, and concurrent myomectomy was not planned. Induction of labor failed to establish active labor, and CD was subsequently performed after counseling and informed consent.

### Intraoperative findings and procedure

A lower transverse uterine incision was made. Upon opening the uterine cavity, a large anterior intramural leiomyoma (FIGO type 2–3) was identified occupying the lower uterine segment and protruding directly into the hysterotomy line. The male infant weighing 2,480 g was delivered without immediate complication, with Apgar scores of 9 and 10 at 1 and 5 min, respectively. Following fetal delivery, the fibroid was found to mechanically separate the myometrial edges, such that approximation for secure hysterotomy closure was not feasible without prior enucleation.

**Figure 2:**
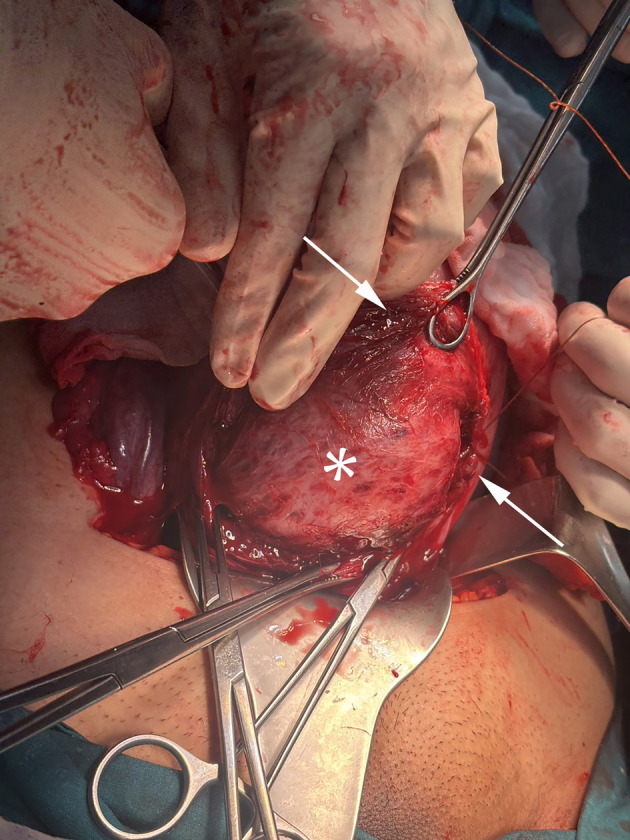
Intraoperative photograph demonstrating the surgical field during trans-endometrial cesarean myomectomy. The upper arrow indicates the point at which the intramural fibroid protrudes into the hysterotomy incision line, preventing myometrial approximation. The asterisk (*) denotes the fibroid body (pseudocapsule surface) following exposure through the existing hysterotomy incision. The lower arrow indicates the myometrial edge, illustrating the anatomical impossibility of uterine closure without prior fibroid enucleation. No additional serosal incision was required.

Trans-endometrial myomectomy was therefore performed through the existing hysterotomy (Fig. 2). In this context, the trans-endometrial approach refers to entry into the fibroid capsule from the internal surface of the pre-existing hysterotomy incision, without creation of any additional serosal or myometrial incision, and is distinct from the trans-serosal approach, which requires a separate external incision. The myoma capsule was entered through the hysterotomy incision, and the lesion was enucleated using combined blunt and sharp dissection. The endometrial lining was partially involved at the myoma bed.

**Figure 3:**
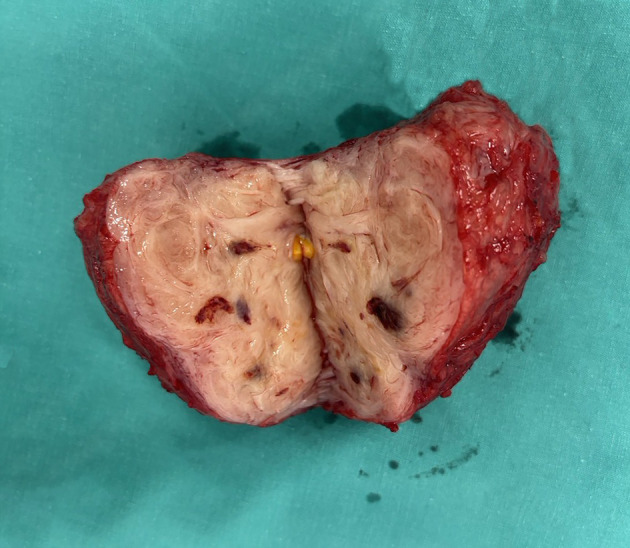
Gross specimen of the excised leiomyoma following trans-endometrial cesarean myomectomy, weighing 239 g. The cut surface demonstrates the characteristic whorled appearance of uterine leiomyoma. Histopathological examination confirmed the diagnosis of leiomyoma.

The myoma bed was repaired separately using interrupted figure-of-eight absorbable sutures prior to hysterotomy closure, incorporating the endometrial defect into this repair. Hemostasis was further supported through a multimodal approach comprising continuous intravenous oxytocin infusion, carbetocin, methylergometrine, and intravenous tranexamic acid, supplemented by warm uterine irrigation. Intrauterine balloon tamponade and temporary uterine artery ligation were considered but were not required. The uterus was then closed in two layers. Estimated blood loss was 1,300 mL, and one unit of packed red blood cells was transfused. Postoperative hemoglobin was 9.7 g/dL. Histopathological examination confirmed leiomyoma, weighing 239 g (Fig. 3). The patient recovered uneventfully and was discharged on postoperative day 3.

### Follow-up and outcomes

At 6 weeks postpartum follow-up, the patient reported no secondary postpartum hemorrhage, fever, or pelvic pain. Physical examination was unremarkable. Transvaginal ultrasonography demonstrated a normal postpartum uterine appearance with no abnormal intrauterine findings.

### Ethical approval

This case report was reviewed and approved as exempt research by the Human Research Ethics Committee of the Faculty of Medicine, Srinakharinwirot University (Approval No. SWUEC683100).

## Discussion

This case highlights a practical role for trans-endometrial CM in a challenging intraoperative situation. A large anterior lower uterine segment intramural leiomyoma (FIGO type 2–3) prevented approximation of the myometrial edges during hysterotomy closure, and myomectomy performed through the existing hysterotomy allowed successful uterine closure. Estimated blood loss was 1,300 mL, and the patient recovered uneventfully with normal transvaginal ultrasonography at 6 weeks postpartum.

Trans-endometrial myomectomy, first described by Hatirnaz et al [10], uses the uterine cavity as the route of access to the fibroid and may avoid an additional serosal incision. In selected cases, prior studies have reported favorable short-term surgical outcomes, including less bleeding and fewer adhesions than with the traditional trans-serosal approach [10–12]. Some data have also suggested acceptable obstetric outcomes in subsequent pregnancies [13]. In this patient, myomectomy was not planned but became an intraoperative technical requirement for surgical completion: the fibroid directly occupied the hysterotomy line and rendered approximation of the myometrial edges anatomically impossible without prior enucleation. The estimated blood loss and any concern regarding scar integrity must therefore be interpreted in the context of a necessary bail-out maneuver rather than an elective procedure. The trans-endometrial approach provided direct access to the fibroid at the incision line and avoided an additional serosal incision [10]. When the endometrial cavity is entered, meticulous hemostasis and multilayer repair are essential.

Meta-analyses have reported higher intraoperative blood loss and longer operative time with CM than with CD alone [6–8]. Hemorrhagic risk varies according to fibroid size, location, and surgical complexity. In the present case, the estimated blood loss was 1,300 mL, highlighting the substantial bleeding risk that may accompany CM when the procedure is required to facilitate uterine closure.

The surgical findings and short-term outcomes in the present case were broadly consistent with those reported in previous studies. Hatirnaz et al [10, 12] described favorable surgical outcomes with the trans-endometrial approach, including reduced bleeding in selected cases. In the present case, blood loss was greater, which may be explained by the larger fibroid size and the unplanned nature of the procedure. The outcome also differed from that reported by Wang et al [11], which may be related to differences in fibroid location, the posterior uterine wall in their report versus the anterior lower uterine segment in the present case. Other outcomes, such as postoperative adhesions, could not be assessed in this case.

At 6 weeks postpartum, the patient reported no secondary hemorrhage, fever, or pelvic pain, and transvaginal ultrasonography demonstrated normal uterine appearance with no abnormal intrauterine findings. Longer-term follow-up is required to assess uterine integrity and reproductive outcomes after trans-endometrial CM, and further studies are needed to define the feasibility and safety of this approach in cases of large lower-segment intramural fibroids.

The transmural nature of the uterine repair in this case has implications for subsequent pregnancy management. An interpregnancy interval of at least 12–18 months is advisable to allow adequate myometrial healing [14]. Additionally, transvaginal ultrasonographic assessment for isthmocele formation at the repair site is recommended prior to attempting conception, as niche development has been associated with abnormal uterine bleeding and may affect subsequent pregnancy outcomes [15]. Given that the endometrial cavity was breached during fibroid enucleation, a planned CD in any subsequent pregnancy may be considered, in view of the potential risk of placenta accreta spectrum and of uterine scar dehiscence or rupture in a subsequent pregnancy [16–18]. Delivery at 37 + 0/7 to 38 + 6/7 weeks may be appropriate, with individualization according to the extent and complexity of the prior uterine surgery [19]. However, these recommendations are extrapolated from related surgical contexts, as prospective data specific to trans-endometrial CM remain limited.

### Conclusion

In cases where a lower-segment intramural leiomyoma prevents approximation of the myometrial edges following CD, intraoperative myomectomy may become an unplanned but required step to achieve definitive uterine closure. The trans-endometrial approach, performed through the existing hysterotomy without a separate serosal incision, may be a feasible and practical option in selected cases. Preoperative anticipation of this scenario, preparedness for hemorrhagic risk, and a structured multimodal hemostatic strategy are important considerations when CM is planned or encountered intraoperatively.

### Learning points

Trans-endometrial CM may be a feasible bail-out technique when a large lower-segment intramural fibroid prevents secure hysterotomy closure.

Meticulous repair of the myoma bed prior to hysterotomy closure is essential to minimize blood loss and optimize uterine scar integrity.

Patients undergoing trans-endometrial CM require counseling regarding interpregnancy interval, isthmocele surveillance, and planned elective CD in subsequent pregnancies.

## Data Availability

The authors declare that data supporting the findings of this study are available within the article.

## Abbreviations

CD: cesarean delivery
CM: cesarean myomectomy
FGR: fetal growth restriction
